# *cis*-Bromido(methyl­amine)­bis­(propane-1,3-di­amine)­cobalt(III) dibromide

**DOI:** 10.1107/S160053681301516X

**Published:** 2013-06-12

**Authors:** P. S. Kannan, A. S. Ganeshraja, K. Anbalagan, E. Govindan, A. SubbiahPandi

**Affiliations:** a Department of Physics, S.M.K. Fomra Institute of Technology, Thaiyur, Chennai 603 103, India; b Department of Chemistry, Pondicherry University, Pondicherry 605 014, India; c Department of Physics, Presidency College (Autonomous), Chennai 600 005, India

## Abstract

In the title compound, [CoBr(CH_5_N)(C_3_H_10_N_2_)_2_]Br_2_, the cobalt^III^ ion has a distorted octa­hedral coordination environment and is surrounded by four N atoms in the equatorial plane, with an additional N atom and the Br atom occupying the axial positions. In the crystal, the complex cation and the two counter anions are linked *via* N—H⋯Br and C—H⋯Br hydrogen bonds, forming a three-dimensional network.

## Related literature

In the synthesis of cobalt(III) complexes, substituting an amino ligand for the MeNH_2_ moiety can yield complexes of similar structure, but with differing electron-transfer rates, see: Anbalagan (2011 ▶); Anbalagan *et al.* (2011 ▶). For the biological activity and potential applications of mixed-ligand cobalt(III) complexes, see: Arslan *et al.* (2009 ▶); Delehanty *et al.* (2008 ▶); Sayed *et al.* (1992 ▶); Teicher *et al.* (1990 ▶); Chang *et al.* (2010 ▶). For related structures, see: Anbalagan *et al.* (2009 ▶); Lee *et al.* (2007 ▶); Ramesh *et al.* (2008 ▶); Ravichandran *et al.* (2009 ▶). For Co—N bond lengths, see: Maheshwaran *et al.* (2013 ▶). 
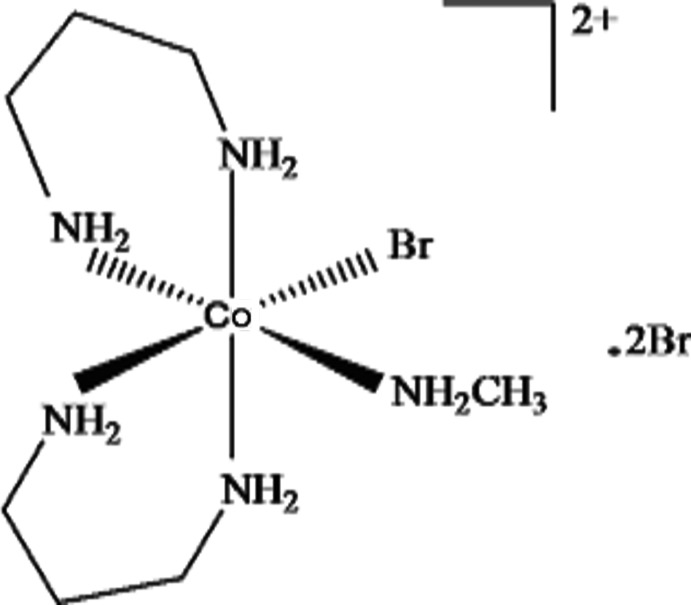


## Experimental

### 

#### Crystal data

[CoBr(CH_5_N)(C_3_H_10_N_2_)_2_]Br_2_
*M* *_r_* = 477.95
Monoclinic, 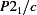

*a* = 13.4418 (2) Å
*b* = 8.3088 (1) Å
*c* = 15.1538 (2) Å
β = 110.61 (2)°
*V* = 1584.16 (4) Å^3^
*Z* = 4
Mo *K*α radiation
μ = 8.64 mm^−1^
*T* = 293 K
0.25 × 0.22 × 0.19 mm

#### Data collection

Oxford Diffraction Xcalibur Eos diffractometer
Absorption correction: multi-scan (*CrysAlis PRO*; Oxford Diffraction, 2009 ▶) *T*_min_ = 0.133, *T*_max_ = 0.194
6000 measured reflections
2784 independent reflections
1701 reflections with *I* > 2σ(*I*)
*R*_int_ = 0.042

#### Refinement

*R*[*F* ^2^ > 2σ(*F* ^2^)] = 0.073
*wR*(*F* ^2^) = 0.238
*S* = 1.07
2784 reflections
146 parameters
H-atom parameters constrained
Δρ_max_ = 1.50 e Å^−3^
Δρ_min_ = −2.69 e Å^−3^

### 

Data collection: *CrysAlis CCD* (Oxford Diffraction, 2009 ▶); cell refinement: *CrysAlis CCD*; data reduction: *CrysAlis RED* (Oxford Diffraction, 2009 ▶); program(s) used to solve structure: *SHELXS97* (Sheldrick, 2008 ▶); program(s) used to refine structure: *SHELXL97* (Sheldrick, 2008 ▶); molecular graphics: *ORTEP-3 for Windows* (Farrugia, 2012 ▶); software used to prepare material for publication: *SHELXL97* and *PLATON* (Spek, 2009 ▶).

## Supplementary Material

Crystal structure: contains datablock(s) global, I. DOI: 10.1107/S160053681301516X/rn2115sup1.cif

Structure factors: contains datablock(s) I. DOI: 10.1107/S160053681301516X/rn2115Isup2.hkl

Additional supplementary materials:  crystallographic information; 3D view; checkCIF report

## Figures and Tables

**Table 1:** Hydrogen-bond geometry (Å, °)

*D*—H⋯*A*	*D*—H	H⋯*A*	*D*⋯*A*	*D*—H⋯*A*
N1—H1*C*⋯Br2^i^	0.90	2.67	3.489 (11)	152
N1—H1*D*⋯Br2	0.90	2.61	3.504 (10)	174
N2—H2*C*⋯Br3	0.90	2.66	3.526 (10)	162
N2—H2*D*⋯Br3^ii^	0.90	2.64	3.419 (10)	146
N3—H3*C*⋯Br3	0.90	2.53	3.406 (12)	164
N3—H3*D*⋯Br2	0.90	2.59	3.482 (11)	171
N4—H4*C*⋯Br2^iii^	0.90	2.62	3.511 (10)	170
N4—H4*D*⋯Br3^ii^	0.90	2.49	3.379 (11)	170
N5—H5*C*⋯Br2^iii^	0.90	2.77	3.632 (11)	160
N5—H5*D*⋯Br2^i^	0.90	2.64	3.532 (12)	170
C6—H6*A*⋯Br3^iii^	0.97	2.91	3.773 (16)	148
C7—H7*B*⋯Br1^iv^	0.96	2.90	3.766 (13)	150

Symmetry codes: (i) 
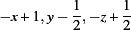
; (ii) 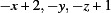; (iii) 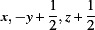; (iv) 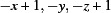.

**Fig. 1.**
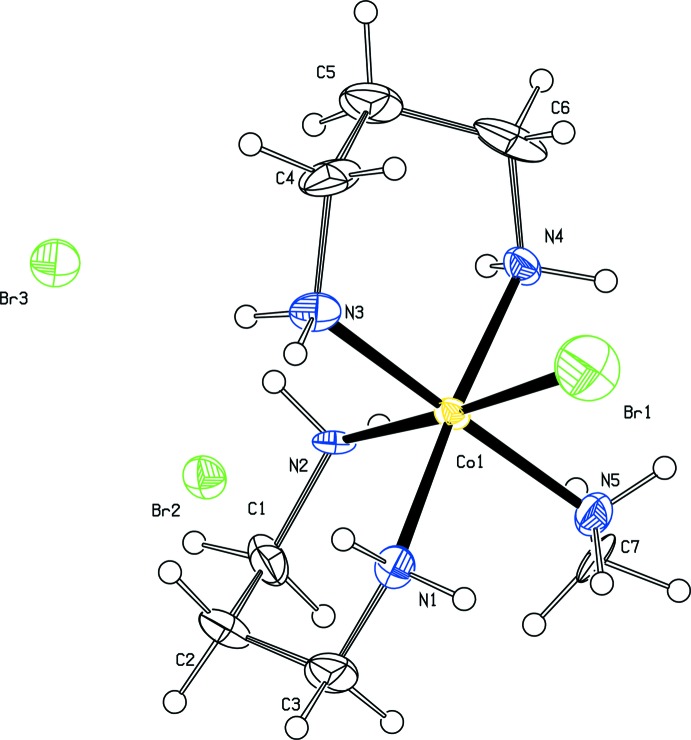
View of the title molecule with the atom labelling scheme. The displacement ellipsoids are drawn at the 30% probability level while the H atoms are shown as small spheres of arbitrary radii.

**Fig. 2.**
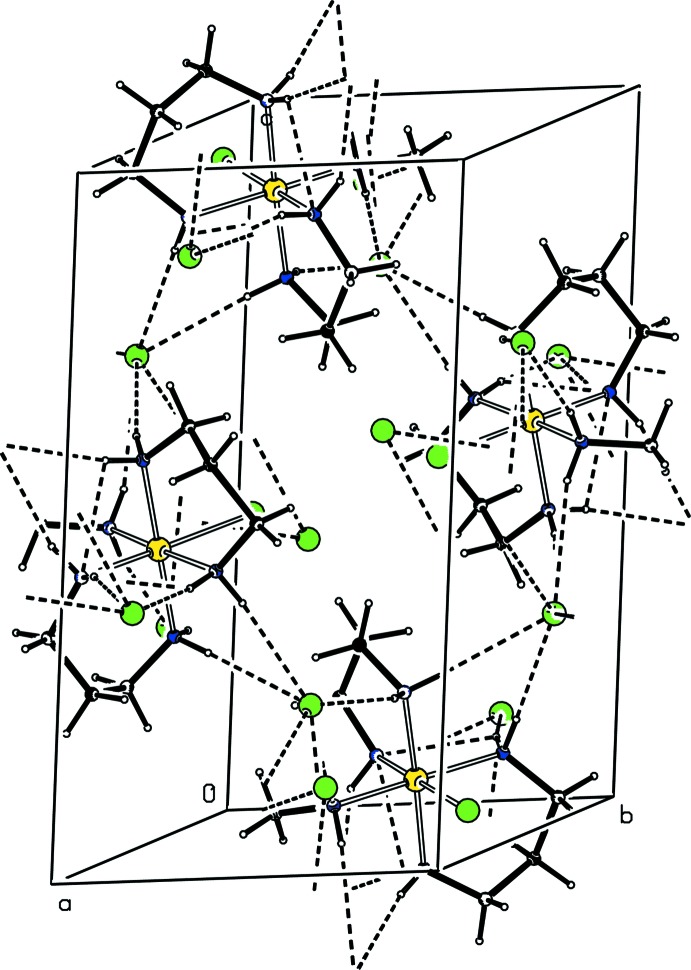
The molecular packing viewed down the a axis. Dashed lines shows the intermolecular N-H···Br and C-H···Br hydrogen bonds.

## supplementary crystallographic information

### Comment

Mixed ligand cobalt(III) complexes find potential applications in the fields of antitumor, antibacterial, antimicrobial, radiosenzitation and cytotoxicity activities (Sayed *et al.*, 1992; Teicher *et al.*, 1990; Arslan *et al.*, 2009; Delehanty *et al.*, 2008). Cobalt is an essential and integral component of vitamin B12, therefore it is physiologically found in most tissues. Complexes of cobalt are useful for nutritional supplementation to provide cobalt in a form which effectively increases the bioavailability, for instance, vitamin B12 by microorganisms present in the gut. In addition, cobalt(III) complexes are known for electron transfer and ligand substitution reactions, which find applications in chemical and biological systems. Against this background and to ascertain the molecular conformation, the structure determination of the title compound has been carried out.

The present research is the design and synthesis of cobalt(III) complexes with an objective to understand the structure-reactivity correlation. Substituting an amino ligand for the MeNH_2_ moiety can yield complexes of similar structure, but with differing electron transfer rates (Anbalagan, 2011; Anbalagan *et al.*, 2011).

X-ray analysis confirms the molecular structure and atom connectivity as illustrated in Fig. 1. The bond lengths [Co-N] in (Fig. 1) agree with those observed [1.9722 (2) to 1.988 (2)Å] in the literature (Maheshwaran *et al.* (2013). The whole molecule is not planar as the dihedral angle between the two pyrimidine rings is 84.8 (5)°. The bond lengths [Co-N] are comparable with the values reported[1.9493 (1) to 1.9673 (2)Å]in the literature (Lee *et al.*, 2007; Ramesh *et al.*, 2008; Anbalagan *et al.*, 2009; Ravichandran *et al.*,2009). One of the six membered rings in the molecule adopts a chair conformation. The crystal packing is stabilized by C–H···Br and N–H···Br interactions along the *a* axis as shown in Fig.2.

### Experimental

Crystalline trans-[Co^III^(tn)_2_Br_2_]Br (2g) was made into a paste using 3-4 drops of water. To the solid mass, about 4 ml of 0.12 M methyl amine (MeNH_2_) was dropped for 30 min and mixed well. The grinding of a dull green paste was continued to obtain a red mass and the reaction mixture was set aside until no further change was observed. Then the product was allowed to stand overnight and the solid was washed with ethanol. The final product was dissolved in 5-10 ml of water pre-heated to 70°C and allowed to crystallize in hot acidified water(few drops of hot conc. HCl and 2 ml of water and cooled). Finally, microcrystalline pink color crystals were retrieved (yield 0.87 g), filtered, washed with ethanol and dried over vacuum. X-ray quality crystals were obtained by recrystallization from hot acidified distilled water.

### Refinement

All H atoms were fixed geometrically and allowed to ride on their parent C atoms, with C—H distances fixed in the range 0.93–0.97 Å with U_iso_(H) = 1.5U_eq_(C) for methyl H 1.2U_eq_(C) for other H atoms.

### Crystal data

**Table:**

[CoBr(CH_5_N)(C_3_H_10_N_2_)_2_]Br_2_	*F*(000) = 936
*M**_r_* = 477.95	*D*_x_ = 2.004 Mg m^−^^3^
Monoclinic, *P*2_1_/*c*	Mo *K*α radiation, λ = 0.71073 Å
Hall symbol: P 2ybc	Cell parameters from 2784 reflections
*a* = 13.4418 (2) Å	θ = 2.8–25.0°
*b* = 8.3088 (1) Å	µ = 8.64 mm^−^^1^
*c* = 15.1538 (2) Å	*T* = 293 K
β = 110.61 (2)°	Block, pink
*V* = 1584.16 (4) Å^3^	0.25 × 0.22 × 0.19 mm
*Z* = 4	

### Data collection

**Table:**

Oxford Diffraction Xcalibur Eos diffractometer	2784 independent reflections
Radiation source: fine-focus sealed tube	1701 reflections with *I* > 2σ(*I*)
Graphite monochromator	*R*_int_ = 0.042
ω and φ scans	θ_max_ = 25.0°, θ_min_ = 2.8°
Absorption correction: multi-scan (*CrysAlis PRO*; Oxford Diffraction, 2009)	*h* = −15→15
*T*_min_ = 0.133, *T*_max_ = 0.194	*k* = −9→9
6000 measured reflections	*l* = −18→18

### Refinement

**Table:**

Refinement on *F*^2^	Primary atom site location: structure-invariant direct methods
Least-squares matrix: full	Secondary atom site location: difference Fourier map
*R*[*F*^2^ > 2σ(*F*^2^)] = 0.073	Hydrogen site location: inferred from neighbouring sites
*wR*(*F*^2^) = 0.238	H-atom parameters constrained
*S* = 1.07	*w* = 1/[σ^2^(*F*_o_^2^) + (0.1435*P*)^2^] where *P* = (*F*_o_^2^ + 2*F*_c_^2^)/3
2784 reflections	(Δ/σ)_max_ < 0.001
146 parameters	Δρ_max_ = 1.50 e Å^−^^3^
0 restraints	Δρ_min_ = −2.69 e Å^−^^3^

### Special details

**Table:**

Geometry. All esds (except the esd in the dihedral angle between two l.s. planes) are estimated using the full covariance matrix. The cell esds are taken into account individually in the estimation of esds in distances, angles and torsion angles; correlations between esds in cell parameters are only used when they are defined by crystal symmetry. An approximate (isotropic) treatment of cell esds is used for estimating esds involving l.s. planes.
Refinement. Refinement of F^2^ against ALL reflections. The weighted R-factor wR and goodness of fit S are based on F^2^, conventional R-factors R are based on F, with F set to zero for negative F^2^. The threshold expression of F^2^ > 2sigma(F^2^) is used only for calculating R-factors(gt) etc. and is not relevant to the choice of reflections for refinement. R-factors based on F^2^ are statistically about twice as large as those based on F, and R- factors based on ALL data will be even larger.

### Fractional atomic coordinates and isotropic or equivalent isotropic displacement parameters (Å^2^)

**Table:**

	*x*	*y*	*z*	*U*_iso_*/*U*_eq_	
C1	0.7910 (12)	−0.1257 (17)	0.3267 (9)	0.045 (4)	
H1A	0.7559	−0.2250	0.3325	0.054*	
H1B	0.8575	−0.1541	0.3190	0.054*	
C2	0.7216 (13)	−0.038 (2)	0.2389 (10)	0.056 (4)	
H2A	0.7566	0.0620	0.2334	0.067*	
H2B	0.7157	−0.1030	0.1842	0.067*	
C3	0.6107 (12)	0.0016 (18)	0.2366 (10)	0.050 (4)	
H3A	0.5682	0.0407	0.1744	0.060*	
H3B	0.5774	−0.0953	0.2488	0.060*	
C4	0.8425 (12)	0.4274 (18)	0.4830 (12)	0.057 (4)	
H4A	0.7892	0.4891	0.4979	0.068*	
H4B	0.8831	0.5004	0.4588	0.068*	
C5	0.9195 (13)	0.336 (2)	0.5754 (11)	0.058 (5)	
H5A	0.9642	0.2605	0.5579	0.070*	
H5B	0.9651	0.4139	0.6189	0.070*	
C6	0.8508 (14)	0.245 (2)	0.6238 (11)	0.066 (5)	
H6A	0.8962	0.2131	0.6866	0.079*	
H6B	0.7977	0.3187	0.6305	0.079*	
C7	0.6526 (9)	−0.2195 (12)	0.4742 (10)	0.032 (3)	
H7A	0.6497	−0.2628	0.4146	0.047*	
H7B	0.6053	−0.2787	0.4970	0.047*	
H7C	0.7238	−0.2280	0.5186	0.047*	
N1	0.6135 (8)	0.1214 (12)	0.3058 (7)	0.030 (2)	
H1C	0.5473	0.1264	0.3074	0.036*	
H1D	0.6251	0.2161	0.2822	0.036*	
N2	0.8141 (7)	−0.0346 (11)	0.4126 (7)	0.027 (2)	
H2C	0.8726	0.0238	0.4188	0.033*	
H2D	0.8332	−0.1067	0.4599	0.033*	
N3	0.7916 (8)	0.3030 (13)	0.4129 (8)	0.036 (3)	
H3C	0.8430	0.2608	0.3947	0.043*	
H3D	0.7466	0.3545	0.3623	0.043*	
N4	0.7983 (7)	0.1060 (13)	0.5734 (7)	0.032 (3)	
H4C	0.7564	0.0679	0.6037	0.038*	
H4D	0.8488	0.0312	0.5795	0.038*	
N5	0.6215 (8)	−0.0543 (14)	0.4628 (8)	0.040 (3)	
H5C	0.6091	−0.0255	0.5152	0.047*	
H5D	0.5584	−0.0503	0.4151	0.047*	
Co1	0.71072 (12)	0.11628 (19)	0.43861 (10)	0.0226 (5)	
Br1	0.59629 (16)	0.3048 (2)	0.47361 (15)	0.0770 (7)	
Br2	0.63730 (10)	0.49197 (15)	0.20461 (9)	0.0347 (4)	
Br3	1.01970 (11)	0.19252 (18)	0.37939 (10)	0.0427 (5)	

### Atomic displacement parameters (Å^2^)

**Table:**

	*U*^11^	*U*^22^	*U*^33^	*U*^12^	*U*^13^	*U*^23^
C1	0.066 (10)	0.044 (9)	0.038 (8)	−0.001 (8)	0.033 (8)	−0.007 (7)
C2	0.068 (11)	0.070 (11)	0.026 (8)	−0.016 (9)	0.014 (8)	−0.020 (8)
C3	0.047 (9)	0.066 (11)	0.023 (7)	−0.011 (8)	−0.003 (7)	0.002 (8)
C4	0.054 (10)	0.040 (9)	0.068 (12)	−0.025 (8)	0.012 (9)	−0.021 (9)
C5	0.045 (9)	0.076 (12)	0.035 (9)	−0.002 (9)	−0.009 (8)	−0.009 (9)
C6	0.076 (12)	0.074 (11)	0.024 (8)	0.019 (10)	−0.012 (8)	−0.017 (8)
C7	0.021 (6)	0.008 (6)	0.074 (10)	−0.004 (5)	0.028 (7)	0.001 (6)
N1	0.026 (6)	0.036 (6)	0.025 (6)	0.004 (5)	0.004 (5)	0.012 (5)
N2	0.027 (6)	0.026 (5)	0.022 (6)	−0.008 (5)	0.001 (5)	−0.011 (5)
N3	0.023 (5)	0.043 (6)	0.030 (6)	0.006 (5)	−0.006 (5)	0.003 (6)
N4	0.023 (5)	0.050 (7)	0.020 (5)	0.003 (5)	0.006 (4)	−0.004 (5)
N5	0.025 (6)	0.062 (8)	0.030 (6)	−0.009 (6)	0.008 (5)	0.005 (6)
Co1	0.0207 (9)	0.0284 (9)	0.0152 (8)	0.0014 (7)	0.0021 (7)	0.0000 (7)
Br1	0.0685 (13)	0.0820 (14)	0.0784 (15)	0.0148 (10)	0.0233 (11)	0.0000 (11)
Br2	0.0320 (8)	0.0419 (8)	0.0268 (7)	0.0045 (6)	0.0061 (6)	0.0049 (6)
Br3	0.0342 (8)	0.0514 (10)	0.0363 (9)	0.0058 (7)	0.0046 (6)	0.0074 (7)

### Geometric parameters (Å, º)

**Table:**

C1—N2	1.442 (15)	C7—N5	1.427 (15)
C1—C2	1.52 (2)	C7—H7A	0.9600
C1—H1A	0.9700	C7—H7B	0.9600
C1—H1B	0.9700	C7—H7C	0.9600
C2—C3	1.51 (2)	N1—Co1	1.977 (9)
C2—H2A	0.9700	N1—H1C	0.9000
C2—H2B	0.9700	N1—H1D	0.9000
C3—N1	1.436 (17)	N2—Co1	2.012 (9)
C3—H3A	0.9700	N2—H2C	0.9000
C3—H3B	0.9700	N2—H2D	0.9000
C4—N3	1.467 (16)	N3—Co1	2.010 (11)
C4—C5	1.61 (2)	N3—H3C	0.9000
C4—H4A	0.9700	N3—H3D	0.9000
C4—H4B	0.9700	N4—Co1	1.967 (9)
C5—C6	1.56 (2)	N4—H4C	0.9000
C5—H5A	0.9700	N4—H4D	0.9000
C5—H5B	0.9700	N5—Co1	1.973 (10)
C6—N4	1.429 (18)	N5—H5C	0.9000
C6—H6A	0.9700	N5—H5D	0.9000
C6—H6B	0.9700	Co1—Br1	2.383 (2)
			
N2—C1—C2	114.2 (12)	Co1—N1—H1C	106.2
N2—C1—H1A	108.7	C3—N1—H1D	106.2
C2—C1—H1A	108.7	Co1—N1—H1D	106.2
N2—C1—H1B	108.7	H1C—N1—H1D	106.4
C2—C1—H1B	108.7	C1—N2—Co1	124.1 (8)
H1A—C1—H1B	107.6	C1—N2—H2C	106.3
C3—C2—C1	114.9 (12)	Co1—N2—H2C	106.3
C3—C2—H2A	108.6	C1—N2—H2D	106.3
C1—C2—H2A	108.6	Co1—N2—H2D	106.3
C3—C2—H2B	108.6	H2C—N2—H2D	106.4
C1—C2—H2B	108.6	C4—N3—Co1	123.3 (9)
H2A—C2—H2B	107.5	C4—N3—H3C	106.5
N1—C3—C2	111.1 (11)	Co1—N3—H3C	106.5
N1—C3—H3A	109.4	C4—N3—H3D	106.5
C2—C3—H3A	109.4	Co1—N3—H3D	106.5
N1—C3—H3B	109.4	H3C—N3—H3D	106.5
C2—C3—H3B	109.4	C6—N4—Co1	121.5 (9)
H3A—C3—H3B	108.0	C6—N4—H4C	107.0
N3—C4—C5	107.0 (12)	Co1—N4—H4C	107.0
N3—C4—H4A	110.3	C6—N4—H4D	107.0
C5—C4—H4A	110.3	Co1—N4—H4D	107.0
N3—C4—H4B	110.3	H4C—N4—H4D	106.7
C5—C4—H4B	110.3	C7—N5—Co1	122.8 (8)
H4A—C4—H4B	108.6	C7—N5—H5C	106.6
C6—C5—C4	109.3 (13)	Co1—N5—H5C	106.6
C6—C5—H5A	109.8	C7—N5—H5D	106.6
C4—C5—H5A	109.8	Co1—N5—H5D	106.6
C6—C5—H5B	109.8	H5C—N5—H5D	106.6
C4—C5—H5B	109.8	N4—Co1—N5	87.5 (4)
H5A—C5—H5B	108.3	N4—Co1—N1	175.7 (4)
N4—C6—C5	113.8 (12)	N5—Co1—N1	88.7 (4)
N4—C6—H6A	108.8	N4—Co1—N3	93.9 (4)
C5—C6—H6A	108.8	N5—Co1—N3	175.2 (4)
N4—C6—H6B	108.8	N1—Co1—N3	89.7 (4)
C5—C6—H6B	108.8	N4—Co1—N2	88.5 (4)
H6A—C6—H6B	107.7	N5—Co1—N2	95.5 (4)
N5—C7—H7A	109.5	N1—Co1—N2	93.9 (4)
N5—C7—H7B	109.5	N3—Co1—N2	89.1 (4)
H7A—C7—H7B	109.5	N4—Co1—Br1	89.7 (3)
N5—C7—H7C	109.5	N5—Co1—Br1	87.1 (3)
H7A—C7—H7C	109.5	N1—Co1—Br1	88.0 (3)
H7B—C7—H7C	109.5	N3—Co1—Br1	88.4 (3)
C3—N1—Co1	124.5 (8)	N2—Co1—Br1	176.8 (3)
C3—N1—H1C	106.2		
			
N2—C1—C2—C3	−63.7 (17)	C7—N5—Co1—Br1	−167.0 (11)
C1—C2—C3—N1	68.7 (16)	C3—N1—Co1—N4	−104 (6)
N3—C4—C5—C6	−70.6 (16)	C3—N1—Co1—N5	−75.2 (11)
C4—C5—C6—N4	72.2 (17)	C3—N1—Co1—N3	109.4 (11)
C2—C3—N1—Co1	−47.0 (15)	C3—N1—Co1—N2	20.3 (11)
C2—C1—N2—Co1	35.9 (16)	C3—N1—Co1—Br1	−162.3 (10)
C5—C4—N3—Co1	54.3 (15)	C4—N3—Co1—N4	−31.1 (11)
C5—C6—N4—Co1	−51.8 (16)	C4—N3—Co1—N5	76 (6)
C6—N4—Co1—N5	−148.1 (11)	C4—N3—Co1—N1	146.5 (11)
C6—N4—Co1—N1	−119 (5)	C4—N3—Co1—N2	−119.5 (11)
C6—N4—Co1—N3	27.3 (11)	C4—N3—Co1—Br1	58.5 (10)
C6—N4—Co1—N2	116.4 (11)	C1—N2—Co1—N4	162.3 (10)
C6—N4—Co1—Br1	−61.0 (11)	C1—N2—Co1—N5	74.9 (10)
C7—N5—Co1—N4	−77.1 (11)	C1—N2—Co1—N1	−14.2 (10)
C7—N5—Co1—N1	105.0 (11)	C1—N2—Co1—N3	−103.8 (10)
C7—N5—Co1—N3	175 (5)	C1—N2—Co1—Br1	−142 (5)
C7—N5—Co1—N2	11.1 (11)		

### Hydrogen-bond geometry (Å, º)

**Table:**

*D*—H···*A*	*D*—H	H···*A*	*D*···*A*	*D*—H···*A*
N1—H1*C*···Br2^i^	0.90	2.67	3.489 (11)	152
N1—H1*D*···Br2	0.90	2.61	3.504 (10)	174
N2—H2*C*···Br3	0.90	2.66	3.526 (10)	162
N2—H2*D*···Br3^ii^	0.90	2.64	3.419 (10)	146
N3—H3*C*···Br3	0.90	2.53	3.406 (12)	164
N3—H3*D*···Br2	0.90	2.59	3.482 (11)	171
N4—H4*C*···Br2^iii^	0.90	2.62	3.511 (10)	170
N4—H4*D*···Br3^ii^	0.90	2.49	3.379 (11)	170
N5—H5*C*···Br2^iii^	0.90	2.77	3.632 (11)	160
N5—H5*D*···Br2^i^	0.90	2.64	3.532 (12)	170
C6—H6*A*···Br3^iii^	0.97	2.91	3.773 (16)	148
C7—H7*B*···Br1^iv^	0.96	2.90	3.766 (13)	150

Symmetry codes: (i) −*x*+1, *y*−1/2, −*z*+1/2; (ii) −*x*+2, −*y*, −*z*+1; (iii) *x*, −*y*+1/2, *z*+1/2; (iv) −*x*+1, −*y*, −*z*+1.
